# Germline or somatic *GPR101* duplication leads to X-linked acrogigantism: a clinico-pathological and genetic study

**DOI:** 10.1186/s40478-016-0328-1

**Published:** 2016-06-01

**Authors:** Donato Iacovazzo, Richard Caswell, Benjamin Bunce, Sian Jose, Bo Yuan, Laura C. Hernández-Ramírez, Sonal Kapur, Francisca Caimari, Jane Evanson, Francesco Ferraù, Mary N. Dang, Plamena Gabrovska, Sarah J. Larkin, Olaf Ansorge, Celia Rodd, Mary L. Vance, Claudia Ramírez-Renteria, Moisés Mercado, Anthony P. Goldstone, Michael Buchfelder, Christine P. Burren, Alper Gurlek, Pinaki Dutta, Catherine S. Choong, Timothy Cheetham, Giampaolo Trivellin, Constantine A. Stratakis, Maria-Beatriz Lopes, Ashley B. Grossman, Jacqueline Trouillas, James R. Lupski, Sian Ellard, Julian R. Sampson, Federico Roncaroli, Márta Korbonits

**Affiliations:** Centre for Endocrinology, Barts and The London School of Medicine, London, EC1M 6BQ UK; Institute of Biomedical and Clinical Science, University of Exeter Medical School, Exeter, EX2 5DW UK; Institute of Medical Genetics, Cardiff University, Cardiff, CF14 4XN UK; Department of Molecular and Human Genetics, Baylor College of Medicine, Houston, TX 77030 USA; Section on Endocrinology and Genetics, Eunice Kennedy Shriver National Institute of Child Health and Human Development (NICHD), NIH, Bethesda, MD 20892 USA; Department of Radiology, Barts Health NHS Trust, London, EC1A 7BE UK; Department of Neuropathology, Nuffield Department of Clinical Neurosciences, University of Oxford, Oxford, OX1 2JD UK; Department of Pediatrics and Child Health, University of Manitoba, Winnipeg, Manitoba R3T 2 N2 Canada; Department of Endocrinology, University of Virginia, Charlottesville, 22908 USA; Endocrinology Service and Experimental Endocrinology Unit, Hospital de Especialidades Centro Médico Nacional Siglo XXI, IMSS, UNAM, Mexico City, 06720 Mexico; Imperial Centre for Endocrinology, Imperial College Healthcare NHS Trust, W12 0HS London, UK; Department of Neurosurgery, University of Duisburg-Essen, Essen, 45141 Germany; Department of Paediatric Endocrinology, University Hospitals Bristol NHS Foundation Trust, Bristol, BS2 8HW UK; Department of Endocrinology and Metabolism, Faculty of Medicine, Hacettepe University, Ankara, 06100 Turkey; Department of Endocrinology, PGIMER, Chandigarh, 160012 India; Department of Pediatric Endocrinology, Princess Margaret Hospital for Children, Subiaco, 6008 Australia; Newcastle University c/o Department of Paediatric Endocrinology, Royal Victoria Infirmary, Newcastle-upon-Tyne, NE1 4LP UK; Department of Pathology, University of Virginia, Charlottesville, 22908 USA; Oxford Centre for Diabetes, Endocrinology and Metabolism, Radcliffe Department of Medicine, University of Oxford, Oxford, OX1 2JD UK; Department of Pathology, Groupement Hospitalier Est, Hospices Civils de Lyon, Bron, 69500 France; Department of Pediatrics, Baylor College of Medicine Houston, Houston, TX 77030 USA; Human Genome Sequencing Center, Baylor College of Medicine Houston, Houston, TX 77030 USA; Texas Children’s Hospital, Houston, TX 77030 USA; Institute of Brain, Behaviour and Mental Health, University of Manchester, Manchester, M13 9PL UK

**Keywords:** XLAG, Gigantism, GPR101, CNV mutation, Pituitary

## Abstract

Non-syndromic pituitary gigantism can result from *AIP* mutations or the recently identified Xq26.3 microduplication causing X-linked acrogigantism (XLAG). Within Xq26.3, *GPR101* is believed to be the causative gene, and the c.924G > C (p.E308D) variant in this orphan G protein-coupled receptor has been suggested to play a role in the pathogenesis of acromegaly.

We studied 153 patients (58 females and 95 males) with pituitary gigantism. *AIP* mutation-negative cases were screened for *GPR101* duplication through copy number variation droplet digital PCR and high-density aCGH. The genetic, clinical and histopathological features of XLAG patients were studied in detail. 395 peripheral blood and 193 pituitary tumor DNA samples from acromegaly patients were tested for *GPR101* variants.

We identified 12 patients (10 females and 2 males; 7.8 %) with XLAG. In one subject, the duplicated region only contained *GPR101*, but not the other three genes in found to be duplicated in the previously reported patients, defining a new smallest region of overlap of duplications. While females presented with germline mutations, the two male patients harbored the mutation in a mosaic state. Nine patients had pituitary adenomas, while three had hyperplasia. The comparison of the features of XLAG, *AIP*-positive and *GPR101*&*AIP*-negative patients revealed significant differences in sex distribution, age at onset, height, prolactin co-secretion and histological features. The pathological features of XLAG-related adenomas were remarkably similar. These tumors had a sinusoidal and lobular architecture. Sparsely and densely granulated somatotrophs were admixed with lactotrophs; follicle-like structures and calcifications were commonly observed. Patients with sporadic of familial acromegaly did not have an increased prevalence of the c.924G > C (p.E308D) *GPR101* variant compared to public databases.

In conclusion, XLAG can result from germline or somatic duplication of *GPR101*. Duplication of *GPR101* alone is sufficient for the development of XLAG, implicating it as the causative gene within the Xq26.3 region. The pathological features of XLAG-associated pituitary adenomas are typical and, together with the clinical phenotype, should prompt genetic testing.

**Electronic supplementary material:**

The online version of this article (doi:10.1186/s40478-016-0328-1) contains supplementary material, which is available to authorized users.

## Introduction

X-linked acrogigantism (XLAG) is a recently identified cause of early-onset pituitary gigantism [1, 2]. The condition often manifests during the first year of life, occurs more frequently in females usually as sporadic disease with only two families reported so far [1]. XLAG patients develop pituitary hyperplasia or mixed somatotroph/lactotroph adenomas both resulting in significant growth hormone (GH) excess [2]. All previously published patients harbor Xq26.3 microduplications encompassing a region of approximately 500Kb [1, 2]. This region contains the locus of the *GPR101* gene, encoding an orphan G protein-coupled receptor (GPCR) that is significantly overexpressed in the pituitary samples of XLAG patients. In addition, the c.924G > C (p.E308D) *GPR101* missense variant was identified in 4.4 % of a series of patients with sporadic acromegaly. This variant was suggested to represent a disease-associated mutation, as it increases cell proliferation and GH release in vitro [1].

The genetic background of non-syndromic gigantism also includes inactivating germline mutations in the aryl hydrocarbon receptor-interacting protein (*AIP*) gene. These mutations are found in 29–50 % of gigantism cases [3–5], causing a low-penetrance disease typically manifesting clinically in the second decade of life, either sporadically or in the setting of Familial Isolated Pituitary Adenoma (FIPA).

In this study, we aimed to evaluate the prevalence of Xq26.3 microduplication, a copy number variation (CNV) gain including *GPR101*, in a large cohort of 153 patients with non-syndromic pituitary gigantism, who were all screened for *AIP* mutations. We provide a detailed clinical and histopathological description of this condition and compare the clinical characteristics of gigantism patients with *GPR101*, *AIP* and without *GPR101*&*AIP* mutations. We also assessed the prevalence of *GPR101* variants in a large series of patients with acromegaly.

## Materials and methods

### Patient selection

The study cohort consisted of 153 patients (58 females and 95 males) diagnosed with pituitary gigantism. Patients were recruited via the International FIPA consortium network (http://www.fipapatients.org/fipaconsortium). Pituitary gigantism was defined as GH excess associated with accelerated growth velocity (> + 2 SDS, standard deviation score) or abnormally tall stature (> + 3 SDS above the normal mean height, i.e. Z-score > +3, or > +2 SDS above the mid-parental height) [3, 6]. Detailed clinical and biochemical data were obtained from the referring physicians. SDS for height and BMI were calculated based on the Centers for Disease Control (CDC) growth charts and, when available, by country-specific growth charts. Other conditions predisposing to pituitary gigantism, including McCune-Albright syndrome, Multiple Endocrine Neoplasia type 1 (MEN1) and Carney complex, were excluded based on the clinical data and, when appropriate, by genetic testing. Two patients from our cohort [7] were previously reported to carry an Xq26.3 microduplication [1, 2]. Additionally, the prevalence of *GPR101* variants was studied in a cohort of 579 acromegaly patients (sporadic and familial) recruited through the FIPA consortium. The study was approved by the local Ethical Committee and informed consent was obtained from all individual participants included in the study.

### Genetic analyses

The *AIP* gene was tested in all patients through Sanger sequencing and, in most patients, by MLPA dosage as well, as previously described [7, 8]. Patients who were not found to carry an *AIP* mutation were screened for *GPR101* duplication through CNV droplet digital PCR (CNV ddPCR) for *GPR101* in leukocyte- or saliva-derived DNA and, in a subset of patients, in DNA isolated from other sources (pituitary, palatine tonsil, skin and buccal cells). When available, DNA samples from both parents of identified XLAG patients were also tested for *GPR101* duplication using the CNV ddPCR. Positive results were confirmed by means of standard and high-density array comparative genomic hybridization (HD-aCGH) and breakpoint junction analysis. Further methodological details are shown in the Additional file 1.

Three hundred and ninety-five leukocyte- and 193 pituitary tumor-derived DNA samples from patients with acromegaly (total number of patients = 579) were tested for the c.924G > C (p.E308D) *GPR101* variant through Sanger sequencing. Leukocyte-derived DNA samples were also tested for the previously reported c.1098C > A (p.D366E) variant [9, 10]. Sanger sequencing of the whole coding region of *GPR101* was also performed in a subset of DNA samples isolated from 42 randomly selected somatotroph adenomas. Primer sequences are available upon request.

### Pathological assessment

Pituitary adenoma tissue from six XLAG patients was available for review and for further studies. Details of the methods for immunohistochemistry, double immunofluorescence and electron microscopy are reported in the Additional file 1. Mitoses were counted in at least 30 fields at the magnification of x40 using a Nikon Eclipse E600 microscope (Nikon UK, Kingston upon Thames, UK) equipped with a Plan Fluor x40/0.75 objective. Quantification of immunoreactions was performed on images taken at the magnification of x20 with a Leica DM5500 microscope (Leica, Wetzlar, Germany) coupled with a Leica DFC295 camera (Leica). Scoring was performed independently by two co-authors (FR and DI). Ki-67 labelling index was evaluated in at least five representative areas counting at least 1000 cells; the value was given as the percentage of positive nuclei. Somatostatin receptor (SSTR) expression was scored as previously described [11], taking into account the localization and extent of the staining [12].

### Statistical analysis

Parametric data are presented as mean ± standard deviation (SD), and nonparametric data as median [interquartile range, IQR]. Normal distribution was assessed using the Shapiro-Wilk test. Data were analyzed through univariate tests (Chi-square, ANOVA and Kruskal-Wallis tests with Bonferroni and Dunn’s post-hoc tests, as appropriate) using the software Prism v5 (GraphPad Software Inc, La Jolla, CA, USA). Significance was set for *P* values <0.05.

## Results

### Genetics and family history

Of the 153 patients, 63 had *AIP* mutations (*AIP*pos, 41.2 %) (27 were sporadic and 36 familial) and 78 patients (*GPR101*&*AIPneg*, 51 %) were negative for both *AIP* mutations and *GPR101* duplication CNV. Twelve patients (10 females, 2 males) with *GPR101* duplication were identified, accounting for 7.8 % of the whole cohort and 17.2 % of females. The duplication appeared to be germline in the ten female patients, while the two male patients harbored the mutation in a mosaic state. In one of these patients (case IX), the duplication was identified by means of the CNV ddPCR in pituitary-, skin- and palatine tonsil-derived but not in leukocyte-, saliva- or buccal cell-derived DNA [13]. In the other case (case VIII), the duplication was found in the pituitary tissue, while saliva-derived DNA tested negative. Analysis of leukocyte-derived DNA in this patient using the CNV ddPCR showed intermediate results between normal and duplicated dosage, suggesting the presence of a heterogeneous blood cell population as a result of somatic mosaicism (Additional file 1: Figure S1).

**Fig. 1:**
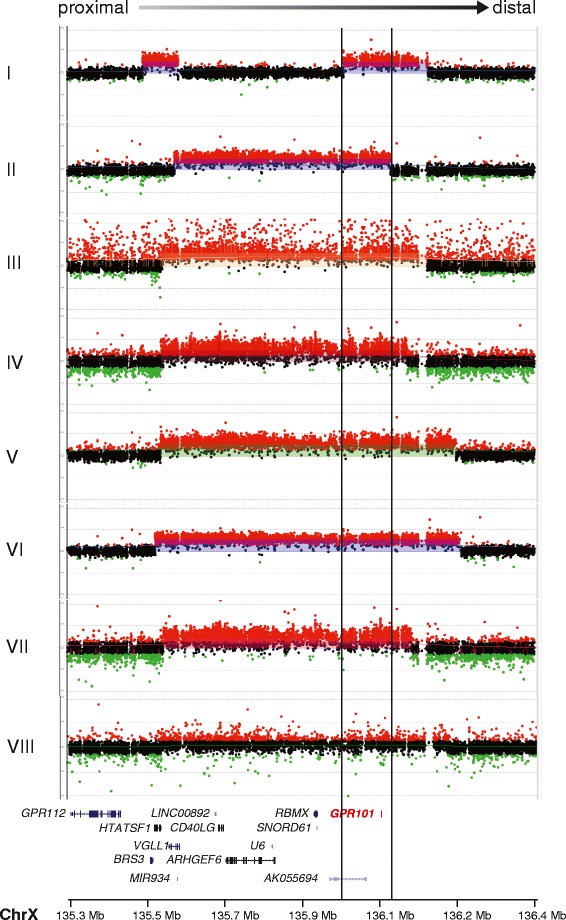
Genomic rearrangements identified in eight subjects with duplications encompassing *GPR101*. The HD-aCGH log_2_ ratio plot for each rearrangement is aligned within the genomic interval of ChrX: 135379766–136479766 (x axis), with the identification of each subject on the y axis. The cluster of red dots shows interrogating oligonucleotide probes that demonstrate increased hybridization intensity, revealing a gain in copy number. The gene content included in the duplicated region is shown underneath the aligned HD-aCGH log_2_ ratio plots. The symbols on the right side of the gene names represent the structure of the genes, with vertical lines representing exons. *GPR101*, a single exon gene, is highlighted in red. The region between the black vertical lines across the log_2_ ratio plots and gene track represent the smallest region of overlap encompassing solely *GPR101*, but not the other three genes (*CD40LG*, *ARHGEF6* and *RBMX*) in the smallest region of overlap of previously published patients. Red probes, log_2_ ratio > 0.25; black probes, −0.25 ≤ log_2_ ratio ≤ 0.25; green probes, log_2_ ratio < −0.25

The CNVs identified in subjects II, III, IV, V, VI, VII and VIII encompassed four genes (*CD40LG*, *ARHGEF6*, *RBMX* and *GPR101*) in the previously reported smallest region of overlap [1]. Remarkably, the distal duplication in case I narrowed down the smallest region of overlap to a genomic region encompassing solely *GPR101* - the putative dosage-sensitive gene responsible for the gigantism trait (Fig. 1). This patient’s duplication was detected by *GPR101* CNV ddPCR and HD-aCGH, while it was not identified with standard aCGH. While most patients’ duplications occurred as a result of the fork stalling and template switching/microhomology-mediated break-induced replication (FoSTeS/MMBIR) mechanism, in one patient (case III) the duplication was generated via an *Alu-Alu* mediated rearrangement (Additional file 1: Table S1). There was no history of pituitary disease in any of these patients’ families. The duplication was found to have occurred de novo in each of the germline mutation cases where DNA samples were available from both parents (4/10 patients).

### Clinical and biochemical features

XLAG patients were generally born at full term and had normal birth length and weight. One patient (case IX) was born large for gestational age (+2.4 SDS). All patients presented with accelerated growth starting as early as seven months of age with a median age at onset of 1.9 years [1.1–2.4]. The median age at diagnosis of gigantism was 4.4 years [2.7–6.7]. Median delay between onset of the disease and diagnosis was 2.6 years [1–3.7]. The median height SDS was +5.4 [4–6.3]. BMI SDS was increased in four out of seven patients with available data, with a median of +2.2 [1.2–3]. Details of symptoms at presentation are shown in the Additional file 1.

Basal GH levels were increased in all patients. IGF-1 levels were increased at 2.9xULN (upper limit of normal) [2–3.9]. Oral glucose tolerance test showed unsuppressed GH (available for 8/12 patients) with a mean change in GH levels of −14.5 % ± 25.5; two patients presented a paradoxical increase of GH levels in response to the glucose load. Patient IV showed a paradoxical rise of GH after thyrotropin-releasing hormone (TRH), and an increase of both GH and prolactin (PRL) levels after the administration of growth hormone-releasing hormone (GHRH) [14]. PRL was elevated in ten patients with a median of 6.9xULN [3.3–11.3]. Circulating GHRH was measured in the three patients with pituitary hyperplasia and was within the reference range. None of the other pituitary axes was affected at diagnosis in any of the patients. No differences in clinical and biochemical parameters were evident between patients carrying germline or somatic *GPR101* duplication.

### Tumor size and extension

Nine of the 12 XLAG patients (75 %) had macroadenomas. Median maximum tumor size was 18 mm [14–25.5]. All the adenomas showed suprasellar extension, and three lesions extended into the cavernous sinus. MRI of three patients (patient II, IV and IX) (25 % of our XLAG cases) showed diffuse enlargement of the gland suggestive of pituitary hyperplasia rather than a distinct adenoma. Representative MRI images are shown in the Additional file 1: Figure S2.

### Histological and immunohistochemical features

**Fig. 2:**
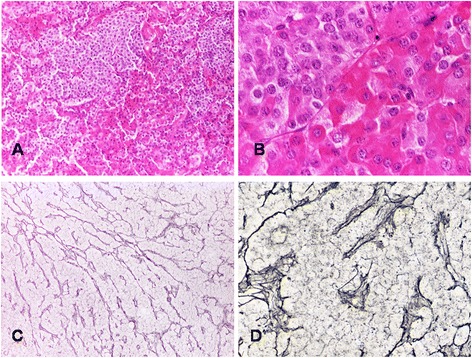
Adenomas occurring in XLAG patients are characterized by distinct populations of acidophilic and chromophobic cells (**a** HE - x10; **b** HE x40); staining for reticulin fibers highlights the lobular and cordonal architecture of XLAG-related adenomas (**c** Gordon-Sweet’s silver impregnation - x10); perivascular connective tissue containing thickened and distorted reticulin fibers (**d** Gordon-Sweet’s silver impregnation – x40)

**Fig. 3:**
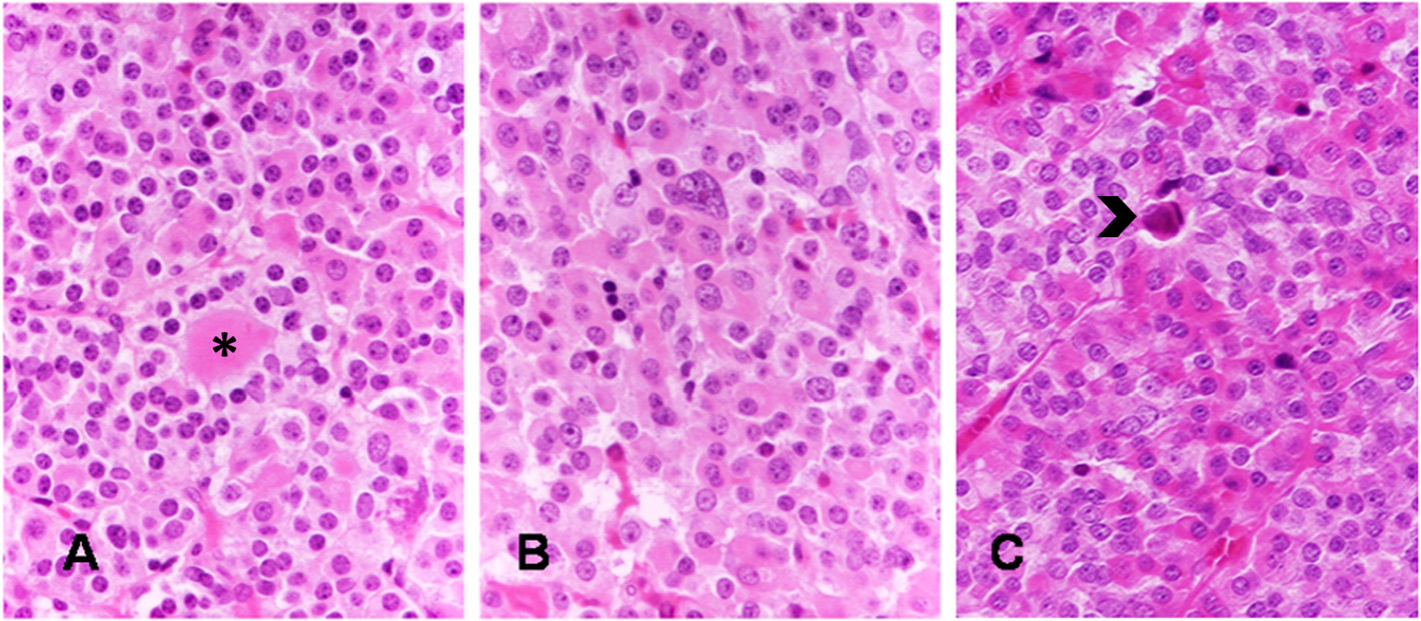
Secondary features of XLAG adenomas include pseudo-follicles containing colloid-like material (asterisk) (**a** HE – x20), isolated cells with large, irregular nucleus (**b** HE – x20), and scattered calcifications (arrow) (**c** HE – x20)

The features of the XLAG-related pituitary adenomas were remarkably similar. They were characterized by a predominantly sinusoidal, lobular and acinar rather than diffuse architecture (Fig. 2a, b). The network of reticulin fibers was disrupted in all tumors although reticulin fibers were still evident in the perivascular connective which appeared stretched and distorted (Fig. 2c, d). Areas of acidophilic cells were admixed with distinct areas of chromophobic cells. Acidophilic cells had large, intensely eosinophilic cytoplasm and centrally placed, rounded nucleus with coarse chromatin and single eosinophilic nucleolus. None of the adenomas investigated in our study contained obvious areas of hyperplasia. Pseudo-follicles containing colloid-like material were noted (Fig. 3a). There was some degree of nuclear pleomorphism (Fig. 3b). Microcalcifications (Fig. 3c) and sparse psammomatous bodies were a frequent feature. Mitotic activity was generally low with an average of one mitosis per 30 high-power fields. None of the adenomas showed necrosis or hemorrhagic changes. One case was adjacent to a Rathke’s cleft cyst.

**Fig. 4:**
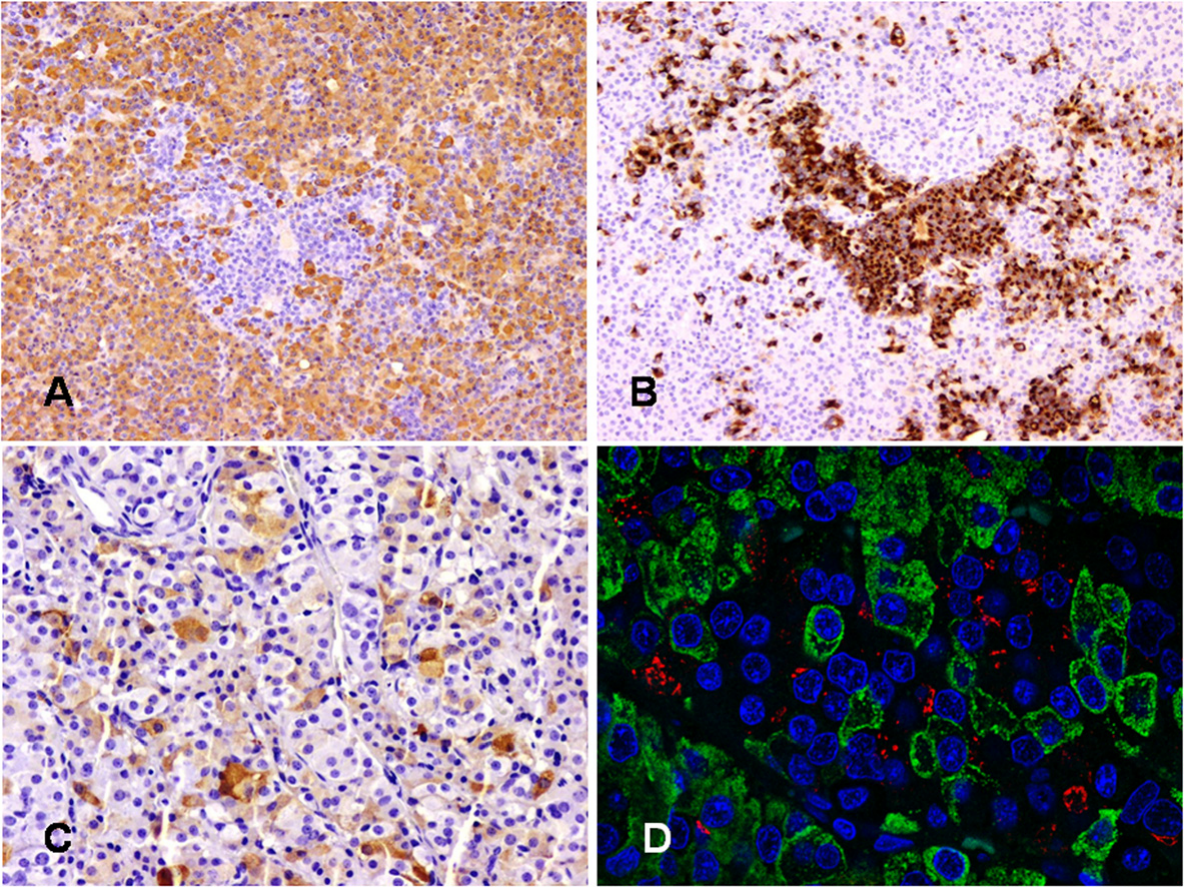
DG somatotrophs represent the predominant component in XLAG adenomas (**a** GH staining with immunoperoxidase – x10), while neoplastic lactotrophs appear as smaller areas (**b** PRL staining, immunoperoxidase – x10); some tumor cells express the common α-subunit (**c** immunoperoxidase – x20); double immunofluorescence for GH (green) and PRL (red) shows lack of co-localization of the two hormones in neoplastic cells (**d** immunofluorescence – x63)

**Fig. 5:**
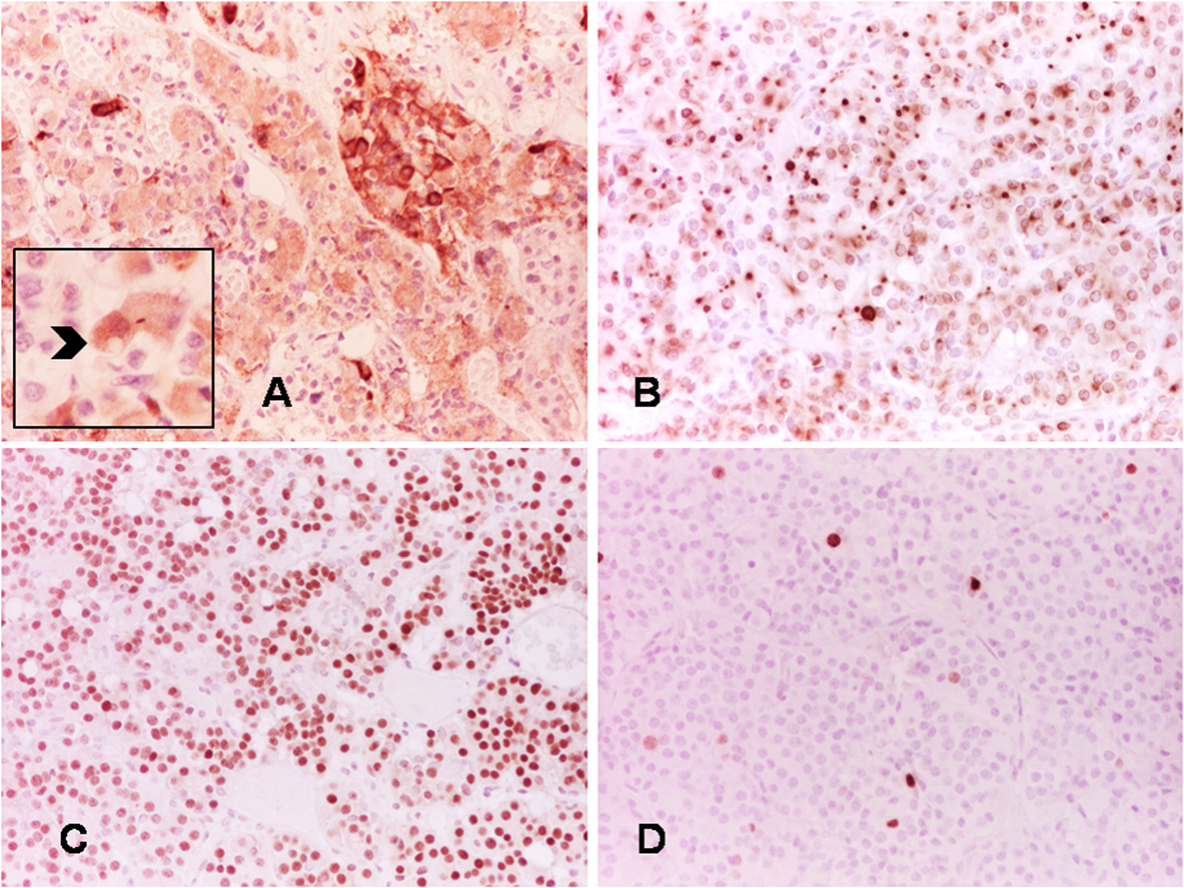
Immunostaining demonstrates areas composed of cells with faint GH expression containing fibrous bodies (arrow) (**a** immunoperoxidase – x20; insert – x40). Fibrous bodies are positive for cytokeratin CAM5.2 (**b** immunoperoxidase – x20); the transcription factor PIT-1 is expressed in the majority of the cells (**c** immunoperoxidase – x20); the Ki-67 labelling index is <3 % in all cases (**d** immunoperoxidase – x20)

On immunohistochemistry, the predominant acidophilic component showed intense positivity for GH, in keeping with densely granulated (DG) somatotrophs (Fig. 4a). Chromophobic cells were less numerous than acidophilic cells accounting for up to approximately 25 % of the whole tumor. The chromophobic component consisted of two cell types. The commonest cells showed large, faintly stained cytoplasm and central nucleus with fine chromatin and inconspicuous nucleolus. These chromophobic cells showed paranuclear positivity for PRL, in keeping with sparsely granulated (SG) lactotrophs (Fig. 4b). Less numerous and mostly interspersed between the acidophilic cells, were slightly smaller chromophobic cells with eccentric nucleus, some of which contained discernible fibrous bodies. These SG somatotroph cells showed weak GH expression (Fig. 5a); fibrous bodies were positive for cytokeratin CAM5.2 (Fig. 5b). One patient (case VIII) had transsphenoidal surgery following radiotherapy. His tumor retained the same features of the other adenomas but it showed more hyperchromatic nuclei and perivascular fibrosis (Additional file 1: Figure S3A). A few mitoses, one of which was atypical, were also present in this case (Additional file 1: Figure S3B).

Scattered adenoma cells showed expression of the common α-subunit (Fig. 4c), while the immunoreactions for ACTH and FSH, LH and TSH β-subunits were negative. Double immunofluorescence highlighted two distinct populations of GH- and PRL-positive cells and no co-localization of the two hormones (Fig. 4d). The transcription factor PIT-1 was expressed in over 90 % of tumor cells (Fig. 5c). The Ki-67 labelling index was lower than 3 % in all cases (Fig. 5d). Weak nuclear p53 expression was noted in all XLAG adenomas, and the degree of expression was similar to sporadic somatotroph adenomas tested as controls. SSTR2a and SSTR5 expression was variable and both cytoplasmic and membranous (scores 1 and 2), although none of the adenomas reached score 3 indicating circumferential membranous staining in more than 50 % of tumor cells. AIP expression was moderate to strong while GHRH immunostaining was negative in all cases. Histological examination of the previously published cases of hyperplasia (case IV and IX) revealed similar features, including enlarged acini with preservation of the reticulin network, substantial increase in somatotroph and lactotroph cells, and scattered cells expressing ACTH and FSH, LH and TSH β-subunits [13, 14].

### Electron microscopy

On electron microscopy, the main cell population in XLAG adenomas was represented by DG cells with well-developed Golgi complexes and rough endoplasmic reticulum containing granules ranging between 250-600 nm in size resembling DG somatotroph cells. Most of the SG cells resembled lactotrophs, showing peripheral granules measuring between 150 and 300 nm and abundant endoplasmic reticulum, while scattered SG somatotroph cells containing fibrous bodies and granules between 200 and 450 nm were also identified (Figure S4). In the hyperplasia cases, a non-tumoral adenohypophysis was seen, dominated by cells with ultrastructural features of somatotrophs and lactotrophs. Many cells containing large pleomorphic secretory granules and exocytosis were identified, showing co-localization of GH and PRL within the same cell and the same granules, as assessed by immunoelectron microscopy in case IV [14], in keeping with mammosomatotroph cells.

### Treatment and outcomes

The clinical features, treatment and outcomes of XLAG patients are detailed in the Additional file 1: Table S2. The median number of treatments per patient was 3.5 [2–4.7]. Five patients received medical therapy as first-line treatment, either with somatostatin analogues (SSAs) combined with dopamine agonists (DAs) (four patients) or DAs alone (one patient), with overall poor results. The GH receptor antagonist pegvisomant was effective in normalizing IGF-1 levels and growth velocity in three of the four patients in whom it was used. Overall, eight out of the 11 patients with surgical or radiotherapeutical intervention developed partial or complete hypopituitarism. Median duration of follow-up was 10.6 years [4.4–22.9]. At the last follow up, overall disease control had been achieved in 11/12 patients, including remission (*n* = 4), disease control (normal age-adjusted IGF-1 in patients on pegvisomant, *n* = 3) and partial control (normal age-adjusted IGF-1 and random GH >1 ng/ml in patients on SSAs and/or DAs, *n* = 4). No other tumors were found to date; the oldest patient with *GPR101* duplication in our series is now 50 years old and has not developed any other disease manifestation.

### Comparison of clinical characteristics of XLAG, *AIP*pos and *GPR101*&*AIP*neg patients

Sex distribution, clinical and biochemical parameters, and MRI findings observed in XLAG patients were compared to those of *AIP*pos and *GPR101*&*AIP*neg patients (Additional file 1: Table S3). Ten of the 12 XLAG patients were females, as opposed to 31.7 % of *AIP*pos and 35.9 % of *GPR101*&*AIP*neg cases (*P* < 0.001 for both comparisons). The median age at onset and diagnosis in XLAG patients was significantly earlier compared to *AIP*pos and *GPR101*&*AIP*neg patients (*P* < 0.001 for both comparisons). The height SDS was also significant higher in XLAG patients compared to *GPR101*&*AIP*neg (*P* < 0.01) and *AIP*pos cases (*P* < 0.05).

No significant difference was found in IGF-1 levels at diagnosis. Hyperprolactinemia was significantly more prevalent in XLAG (83.3 %), compared to *AIP*pos (23.5 %, *P* < 0.001) and *GPR101*&*AIP*neg patients (32.3 %, *P* < 0.01). Median maximum tumor diameter and proportion of macroadenomas was not significantly different in the three groups, although no giant adenomas were found in XLAG patients, while they represented 25–30 % of the cases in the other two groups (*P* < 0.05 XLAG vs *GPR101*&*AIP*neg). Pituitary hyperplasia was significantly more common in XLAG patients (25 %), compared to *AIP*pos (2.6 %, *P* < 0.05) and *GPR101*&*AIP*neg cases (none, *P* < 0.01). No difference was found in the rate of suprasellar extension and cavernous sinus invasion. Pituitary apoplexy was not observed in XLAG patients, while it occurred in 14.5 % of *AIP*pos and 2.9 % of *GPR101*&*AIP*neg cases. There was no significant difference in the rate of hypopituitarism and number of treatments.

### *GPR101* variants in acromegaly patients

We sequenced DNA samples from 579 acromegaly patients (395 leukocyte- and 193 pituitary tumor-derived) and identified four patients (0.69 %) harboring the germline *GPR101* c.924G > C (p.E308D) variant. None of these patients had a family history of pituitary adenoma. The allele frequency of the c.924G > C (p.E308D) variant in our series (0.45 %) was similar to that reported in the Exome Aggregation Consortium Database (ExAC) (0.37 %, *P* = 0.69). The c.1098C > A (p.D366E) variant was not identified in any of the 395 leukocyte-derived DNA samples. The full coding region of the *GPR101* gene was sequenced in 42 unselected sporadic somatotroph adenomas. Two common single nucleotide variants were identified: c.370G > T (p.V124L) (rs1190736, minor allele frequency ExAC database 38 %) and c.1127 T > C (p.L376P) (rs5931046, minor allele frequency 17.2 %). No rare or novel variants were found.

## Discussion

In this large international cohort of patients with non-syndromic pituitary gigantism, we identified germline or somatic *GPR101* duplication causing XLAG in 8 % of patients. This frequency, consistent with previously published findings [5], supports the need for testing for *GPR101* duplication in patients with early-onset pituitary gigantism.

Our results provide significant and clinically relevant mechanistic insights on XLAG. Firstly, we provide proof that duplication of *GPR101* is sufficient to cause the XLAG phenotype. HD-aCGH in one of the patients from our series has allowed the definition of a new smallest region of overlap in XLAG patients to an area encompassing *GPR101* only, but not the other three genes (*CD40LG*, *ARHGEF6* and *RBMX*) previously identified in the duplicated region [1, 2, 15, 16]. Furthermore, our results indicate that, as such small duplications cannot be found on a standard aCGH array, alternative diagnostic methods should be employed, such as CNV ddPCR for *GPR101* or HD-aCGH.

Secondly, as recently described [13, 16], XLAG can result from a duplication occurring during an early postzygotic stage, resulting in somatic mosaicism. The only two male subjects in our series were mosaic for the *GPR101* duplication. In one of these patients, the CNV ddPCR did not show the presence of the duplication in leukocyte-derived DNA [13], indicating that, in the presence of a clinical phenotype suggestive of XLAG, analysis of pituitary- or other tissues-derived DNA samples should be considered in order to confirm the diagnosis.

Thirdly, we have described in detail the histopathological features of XLAG adenomas. Most XLAG patients develop mixed somatotroph/lactotroph adenomas that show a characteristic sinusodoidal and lobular architecture and contain both DG and SG somatotroph cells. Microcalcifications and follicle-like structures are commonly observed. Mitotic activity was negligible in five of our cases and the Ki-67 labelling index never exceeded 3 %.

Adenomas observed in patients with XLAG differ from other GH-secreting adenomas. The classification of somatotroph adenomas is based on morphological, ultrastructural and immunophenotypical features, and includes the common DG and SG somatotroph adenomas, mixed somatotroph/lactotroph adenomas [17], as well as the less frequent mammmosomatotroph [17], silent type III [18] and acidophilic stem cell adenomas [19]. Neoplastic cells of DG somatotroph adenomas typically show acidophilic cytoplasm with intense and widespread immunoreactivity for GH. At electron microscopy, cells resemble normal somatotrophs. Other anterior pituitary hormones are often expressed, including PRL, the common α-subunit and β-subunits of FSH, LH and TSH in various combinations. Prolactin immunoreactivity is by far the commonest in DG adenomas and, in fact, about 50 % of acromegaly patients present with signs and symptoms of hyperprolactinemia [20]. SG somatotroph adenomas are composed of smaller cells, have chromophobic cytoplasm with faint and focal immunoreactivity for GH, reflecting the paucity of secretory granules seen at electron microscopy. SG adenomas contain distinctive paranuclear cytokeratin-positive inclusions known as fibrous bodies. Immunoreactivity for other pituitary hormones is considerably less common than DG adenomas and, when present, their expression is weak. In about 25 % of somatotroph adenomas, tumor cells show intermediate features between DG and SG cells [21], a feature we did not observe in XLAG. In mixed somatotroph/lactotroph adenomas, the degree of hormone expression and distribution of GH and PRL-positive cells vary among cases. Mixed adenomas contain either DG or SG somatotrophs admixed with lactotroph cells [17, 22]; the coexistence of DG and SG somatotroph cells observed in XLAG-related adenomas has never been formally described to the best of our knowledge.

The distinction between nodular hyperplasia and adenoma is certainly challenging, given the lobular, acinar and sinusoidal/cordonal architecture of XLAG-related adenomas. Several features are, however, in keeping with a neoplastic rather than a hyperplastic process. Firstly, reticulin fibers are almost exclusively limited to perivascular spaces, which appeared thickened, distorted and stretched. Secondly, we are not aware of any description of somatotroph hyperplasia containing SG somatotroph cells [23, 24]. In addition, proliferative activity identified with Ki-67, and the presence of a few mitoses, are further features indicative of an adenoma rather than hyperplasia.

It is important to consider that GH excess was caused by mammosomatroph hyperplasia in two of our XLAG patients [13, 14], while one hyperplasia patient had pure GH excess (case II). Interestingly, none of the adenomas examined in this study demonstrated a transition from hyperplasia to adenoma or showed any tumor cells with GH and PRL co-localization. Sampling may explain the lack of hyperplasia, although we cannot exclude that mammosomatotroph hyperplasia and mixed somatotroph/lactotroph adenomas represent distinct pathological manifestations of XLAG.

The mechanisms underlying the molecular pathogenesis of XLAG are unknown. *GPR101* encodes an orphan GPCR [25]. In mice, GPR101 is expressed throughout the central nervous system, with higher levels observed in the hypothalamus [1, 25]. The finding of increased circulating GHRH levels in some patients with XLAG [1, 2, 26] and in other cases with a phenotype highly suggestive of XLAG [27, 28], suggests that upregulation of hypothalamic GHRH might play a pathogenic role. Notably, adenomatous transformation to a SG somatotroph adenoma was reported in a case of pituitary hyperplasia and ectopic acromegaly due to a pituitary metastasis from a GHRH-secreting neuroendocrine tumor [29]. Mammosomatotroph cells account for a sizeable proportion of the human fetal anterior pituitary and are considered precursors of GH and PRL cells in the adult anterior pituitary [30, 31]. These bi-hormonal cells are frequently found in gigantism [32], while they are rare in adult acromegaly patients [20]. The expansion of mammosomatotrophs observed in XLAG patients with pituitary hyperplasia could potentially result from prenatal exposure to increased GHRH levels. Interestingly, mice transgenic for GHRH develop mammosomatotroph hyperplasia [33], and occurrence of somatotroph adenomas has been described in older animals [34]. Moreover, the pituitary cells from one of our patients were found to normally respond to octreotide and bromocriptine treatment in vitro, despite only a marginal biochemical response in vivo [14], further supporting the role for a stimulatory factor hampering responsiveness to medical treatment. Duplication of *GPR101* might potentially affect the GH axis both at the pituitary and the hypothalamic level. In fact, GPR101 is coupled to the stimulatory G protein [25] and, in GH3 mammosomatotroph cells, overexpression of GPR101 has been found to increase cAMP levels [1], which represents a key factor involved in the regulation of GH secretion and cell proliferation in response to GHRH [35, 36].

Patients with XLAG have a unique clinical phenotype, whether it is a result of a germline or somatic *GPR101* duplication. The disease presents very early; excessive growth started before four years of age and was the presenting feature in all patients. We confirmed that XLAG is more common in females, occurs earlier and is more frequently associated with hyperprolactinemia, compared with gigantism due to *AIP* mutations. Pituitary adenomas in *AIP* mutation carriers are frequently large and invasive, invariably SG and often characterized by a high proliferation index [3, 5, 37]. Considering the marginal overlap we observed between XLAG and *AIP*pos patients in regards to the age at disease onset, in the rare case of young-onset gigantism due to an *AIP* mutation [38], neuroimaging and pathological features can help in the differential diagnosis. Interestingly, two of our youngest *AIP*pos cases (aged 4 and 6) presented with pituitary apoplexy [8, 38], which was not observed among XLAG patients. Moreover, although described in the setting of *AIP*-related pituitary disease [39], hyperplasia is an unusual finding in *AIP*pos cases, occurring only in one patient in our series [3].

XLAG patients frequently require multi-modal treatment. First generation SSAs are usually ineffective, while dopamine agonists effectively control PRL excess in the cases where appropriate doses are used, although they don’t seem to have a significant effect on GH and IGF-1 levels. A combination of surgery and radiation was necessary in most patients, and, in some of them, allowed the control of GH excess with further use of SSAs. Pegvisomant was an effective treatment both (i) in patients whose disease was not controlled despite multi-modal treatment, and (ii) in patients with pituitary hyperplasia, where extensive pituitary surgery was not considered due to the risk of hypopituitarism.

Finally, we investigated the prevalence of *GPR101* variants in a large series of acromegaly patients. The frequency of the previously reported c.924G > C (p.E308D) variant in our cohort was found to be similar to that reported in the ExAC database. No other rare or novel coding variants were identified, either at the germline or somatic level, suggesting that *GPR101* variants do not occur frequently and might not play a significant role in the pathogenesis of acromegaly. These results are in line with recently published studies [10, 40].

## Conclusion

XLAG accounts for a significant proportion of patients with non-syndromic gigantism, and results from either germline or somatic duplications involving *GPR101*, which we have proved to be the causative gene within the Xq26.3 region. Our data suggest that specific testing for *GPR101* duplication and, in some cases, testing of alternative tissue-derived DNA samples, should be employed for the genetic diagnosis of XLAG. Most XLAG patients develop pituitary adenomas showing remarkably similar histopathological features, including sinusoidal and lobular architecture, the presence of both DG and SG somatotrophs admixed with lactotroph cells, follicle-like structures and calcifications. These features, together with the clinical phenotype, should raise the suspicion of XLAG. Further studies are needed to untangle the molecular mechanisms involved in the pathogenesis of this condition.

## Abbreviations

XLAG, X-linked acrogigantism; GH, growth hormone; GPCR, G protein-coupled receptor; AIP, aryl hydrocarbon receptor-interacting protein; FIPA, Familial Isolated Pituitary Adenoma; CNV, copy number variation; MEN1, Multiple Endocrine Neoplasia type 1; ddPCR, droplet digital PCR; aCGH, array comparative genomic hybridization; HD-aCGH, high-density array comparative genomic hybridization; PRL, prolactin; TRH, thyrotropin-relasing hormone; GHRH, growth hormone-releasing hormone; DG, densely granulated; SG, sparsely granulated; SSTR2a, somatostatin receptor 2a; SSTR5, somatostatin receptor 5; SSA, somatostatin analogue; DA, dopamine agonist.

## Additional file

Additional file 1: contains supplementary methods regarding genetic and pathologic studies. Supplementary results on the genetic analyses, patient clinical features and electron microscopy are also provided. (DOCX 11206 kb)
