# Supplementary material for: Germline or somatic GPR101 duplication leads to X-linked acrogigantism: a clinico-pathological and genetic study
Source: Acta Neuropathol Commun. 2016 Jun 1;4:56. doi: 10.1186/s40478-016-0328-1 (PMC4888203; doi:10.1186/s40478-016-0328-1)
Supplement: Additional file 1: — contains supplementary methods regarding genetic and pathologic studies. Supplementary results on the genetic analyses, patient clinical features and electron microscopy are also provided. (DOCX 11206 kb) [file 40478_2016_328_MOESM1_ESM.docx]

**Supplementary Data**

**Supplementary methods**

**Genetic analyses**

Genomic DNA was extracted from peripheral blood leukocytes and saliva using commercially available kits (blood: Illustra DNA Extraction Kit BACC2, GE Healthcare, Little Chalfont, UK; saliva: Oragene-DNA for sample collection and prepIT-L2P for DNA extraction, DNA Genotek, Ontario, Canada). DNA from archival formalin-fixed paraffin-embedded tissue, skin and buccal cells was isolated using the Qiagen QIAamp DNA mini kit (QIAGEN, Limburg, Netherlands) following manufacturer’s instructions.

The copy number variation droplet digital PCR (CNV ddPCR) was performed on the Bio-Rad QX200 system using two different TaqMan assays (Life Technologies, Carlsbad, CA, USA) for the detection of *GPR101* (Hs01818174_cn and Hs01730605_cn, targeting the 5’ and 3’ regions of *GPR101*, respectively). In both assays, *RNaseP* was used as the reference gene. CNV data were calculated by the QX200 Quanta Soft analysis package. Standard array comparative genomic hybridization (aCGH) analysis was carried out using commercial arrays (BlueGnomeCytoChip ISCA 8x60k v2.0, Illumina, San Diego, CA, USA) according to the manufacturer’s instructions. The arrays were scanned with a G2505C microarray scanner (Agilent Technologies, Santa Clara, CA, USA) and analyzed using the BlueFuse Multi (v3.3) software (Illumina) using the manufacturer’s default settings. For high-density (HD) aCGH and breakpoint junction sequencing the experimental procedures followed the published workflow [[1](#_ENREF_1),[2](#_ENREF_2)]. Genomic positions were based on hg19/GRCh37.

**Pathological assessment**

Immunohistochemistry for pituitary hormones, including growth hormone (GH) (Dako, Glostrup, Denmark; rabbit polyclonal, 1:3000), prolactin (PRL) (Dako; rabbit polyclonal, 1:3000), and p53 (Dako; mouse monoclonal, prediluted) was performed by the avidin–biotin method with an automated staining system (Dako Autostainer Plus, Dako). Further stainings were performed using the following primary antibodies directed against: cytokeratin CAM5.2 (Becton Dickinson, Franklin Lakes, NJ, USA; mouse monoclonal, prediluted), Ki-67 (Dako; mouse monoclonal, 1:75), AIP (Novus Biologicals, Littleton, Colorado, USA; mouse monoclonal, dilution 1:1500), somatostatin receptor 2a (SSTR2a) (Abcam, Cambridge, UK; rabbit monoclonal, dilution 1:500), somatostatin receptor 5 (SSTR5) (Abcam; rabbit monoclonal, 1:100), PIT-1 (kindly supplied by Prof Rhodes, Indiana University, Indianapolis, IN, USA; rabbit polyclonal, 1:1000) and growth hormone-releasing hormone (GHRH) (supplied by Dr Cohen, INSERM, Lyon, France; rabbit polyclonal, 1:2000). Four μm sections were dewaxed in xylene and rehydrated in decreasing alcohol solutions to distilled water. Antigen unmasking was performed in a microwave oven for 12 minutes at 650W in 10mM citrate buffer at pH 6. Slides were incubated with primary antibodies for 30 minutes at room temperature. After washing in PBS-Tween 20, they were incubated for 30 minutes with the biotinylated secondary antibody (Vector Laboratories, Burlingame, California, USA). The slides were then washed and the Avidin Biotin Complex (Vector Laboratories) was applied for 30 minutes. The reactions were visualized with DAB peroxidase (Vector Laboratories). Appropriate positive controls were added to each batch. Reactions with omission of the primary antibody were also run as negative controls. The Gordon Sweet silver method was used for the demonstration of reticulin fibers.

*Double immunofluorescence*

Double immunofluorescence was used to study the co-localization of GH and PRL in neoplastic cells. After dewaxing and rehydrating the sections and antigen retrieval in citrate buffer, the slides were incubated for one hour at room temperature with a mix of anti-GH (Santa Cruz Biotechnology, Dallas, TX, USA; mouse monoclonal, dilution 1:100) and anti-PRL (supplied by Dr Parlow, HUMC, Torrance, CA, USA; rabbit polyclonal, dilution 1:100) antibodies. The secondary fluorescent labelled (Life Technologies) goat anti-mouse (Alexa Fluor 488) and goat anti-rabbit (Alexa Fluor 568) antibodies were applied for one hour. Sections were finally rinsed three times before coverslips were applied using fluorescent mounting medium with DAPI (Sigma Aldrich, St. Louis, MO, USA). Images were acquired using the Leica confocal microscope LSM510 (Leica, Wetzlar, Germany). The normal human pituitary served as positive control.

*Electron microscopy*

Electron microscopy (EM) was available in three adenomas and in two cases of pituitary hyperplasia. The tissue was fixed in 3% glutaraldehyde in 0.1M cacodylate buffer and embedded in Spurr’s resin. The semithin sections were stained with toluidine blue to select the most representative areas. Sections were stained with osmium cacodylate and examined with a Hitachi H-7650 electron microscope (Hitachi Maidenhead, Berkshire, UK).

**Supplementary results**

**Genetic analyses**

HD-aCGH and breakpoint junction sequencing were performed on DNA samples from eight subjects. In the DNA samples of patients II, III, IV, V, VI, VII and VIII, HD-aCGH revealed simple duplications (DUP); breakpoint junction sequencing analysis suggested that these DUPs were tandem duplications. In the DNA sample of patient I, HD-aCGH revealed a potential complex genomic rearrangement with a pattern of a proximal duplication sequentially followed by a normal-copy segment and a distal duplication (DUP-normal copy segment-DUP). Breakpoint junction sequencing revealed that the proximal side of the proximal DUP joined the proximal side of the distal DUP in an inverted orientation, representing a complex genomic rearrangement with a DUP-NML/INV-DUP pattern. Microhomologies were identified at the breakpoint junctions of patients I, II, IV, V, VI and VIII, while a 21bp insertion was identified at the breakpoint junction of patient VII (Supplementary Data, Table S1) – these mutational signatures suggest replication-based mechanisms for DNA break repair. These data are consistent with fork stalling and template switching/microhomology-mediated break-induced replication (FoSTeS/MMBIR) as the potential mechanism for the duplication origin in these subjects, as previously suggested [[3-5](#_ENREF_3)]. Notably, in subject III, a new chimeric *Alu* hybrid originating from two directly oriented *Alu*Y elements was observed at the breakpoint junction (Supplementary Data, Table S1), suggesting that this duplication was generated via an *Alu-Alu* mediated rearrangement [[6](#_ENREF_6),[7](#_ENREF_7)].

**Clinical features**

Acral enlargement was reported in eight patients, followed by coarse facial features (five patients), increased appetite (four patients), headaches (three patients), hyperhidrosis (two patients), acanthosis nigricans (two patients), interdental space widening (two patients) and joint pain (one patient). Two patients had *café-au-lait* spots: one patient had two spots on the left arm (round, 1x1 cm) and calf (irregular, 2x2 cm), while the other one had one single spot (round, 1x1 cm) on the foot. None of the patients had visual disturbances or other neurological signs, including clinical signs or symptoms of pituitary apoplexy. Final height was achieved by four of the 12 patients at the last follow-up.

**Treatment and outcomes**

Somatostatin analogues (SSAs) dosage was 20mg of octreotide LAR/4 week, 60mg/4 week lanreotide autogel or 222-1550µg/day of short acting octreotide. Dopamine agonists (DAs) dosage was 7.5mg daily for bromocriptine and 0.3-1mg weekly for cabergoline. In patients receiving SSA treatment GH response was variable with an overall increase of mean GH levels (+52%±160.4). Reduction of GH levels to less than 50% of the baseline was observed in two patients. Mean change of IGF-1 levels was -12.6%±15.2; none of the patients treated with SSAs had greater than 50% drop in IGF-1 levels compared to baseline. DAs reduced PRL levels to less than 50% in three patients and normalized them in two other patients. Tumor volume shrinkage of >20% in response to SSA and/or DA therapy was only observed in one case. Among the nine patients with pituitary macroadenomas, six underwent transsphenoidal surgery and two were operated via a transcranial approach due to a hypopneumatized sphenoid sinus. Post-surgical remission, defined as normal age-adjusted IGF-1 and GH suppression after the oral glucose load to <1ng/ml, was observed in three out of eight patients. Post-operative hypopituitarism occurred in six patients, including the three patients in remission. Five patients received external beam radiotherapy: conventional in one and stereotactic radiosurgery in four cases. Following radiation treatment, the disease was controlled in two patients with further use of SSAs and in another patient with DAs alone. The use of the GH receptor antagonist pegvisomant normalized IGF-1 levels in two other cases. One patient was treated with three intrasellar yttrium implants [[8](#_ENREF_8)]; she later developed hypopituitarism and her disease is now controlled on SSAs. Among the three patients with pituitary hyperplasia, one that was resistant to medical treatment with SSAs and DAs underwent total hypophysectomy [[9](#_ENREF_9)], requiring life-long hormone replacement therapy. One patient had transcranial debulking surgery and is now controlled on the combination of DA, SSA and pegvisomant, and the third patient was resistant to SSA and DA treatment and her disease is now controlled on pegvisomant.

**Supplementary figures and legend**

**
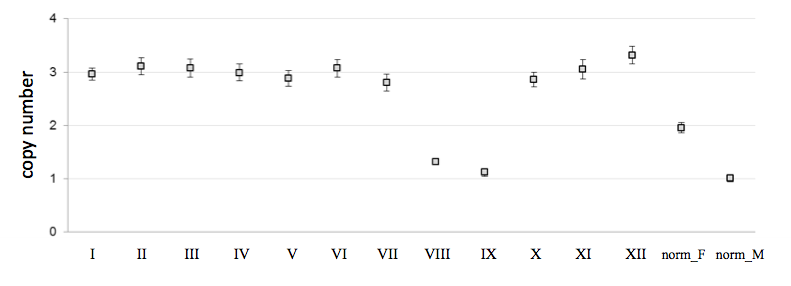
**

**Figure S1** CNV ddPCR results on constitutional DNA samples. Data are displayed as *GPR101* copy number (assay id Hs01818174_cn, Life Technologies) (y axis), relative to the reference gene (*RNaseP*), as calculated by the QuantaSoft analysis software; patient IDs are shown on the x axis. Vertical bars show 95% Poisson confidence limits. norm_f and norm_m show representative results from a normal female and a normal male control, respectively

**
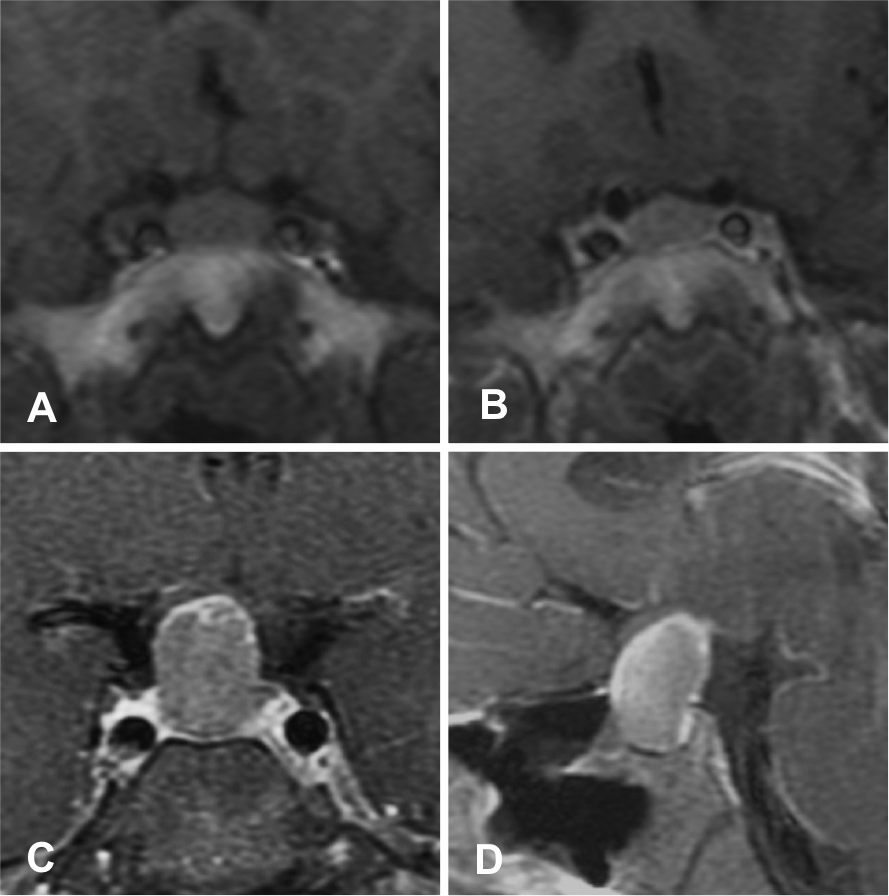
**

**Figure S2** Representative MRI images in XLAG patients. Coronal MRI images from patient II pre- (A) and post-contrast (B) showing diffuse pituitary enlargement without evidence of a distinct adenoma. Coronal (C) and sagittal (D) post-contrast MRI images from patient V showing a macroadenoma with suprasellar extension


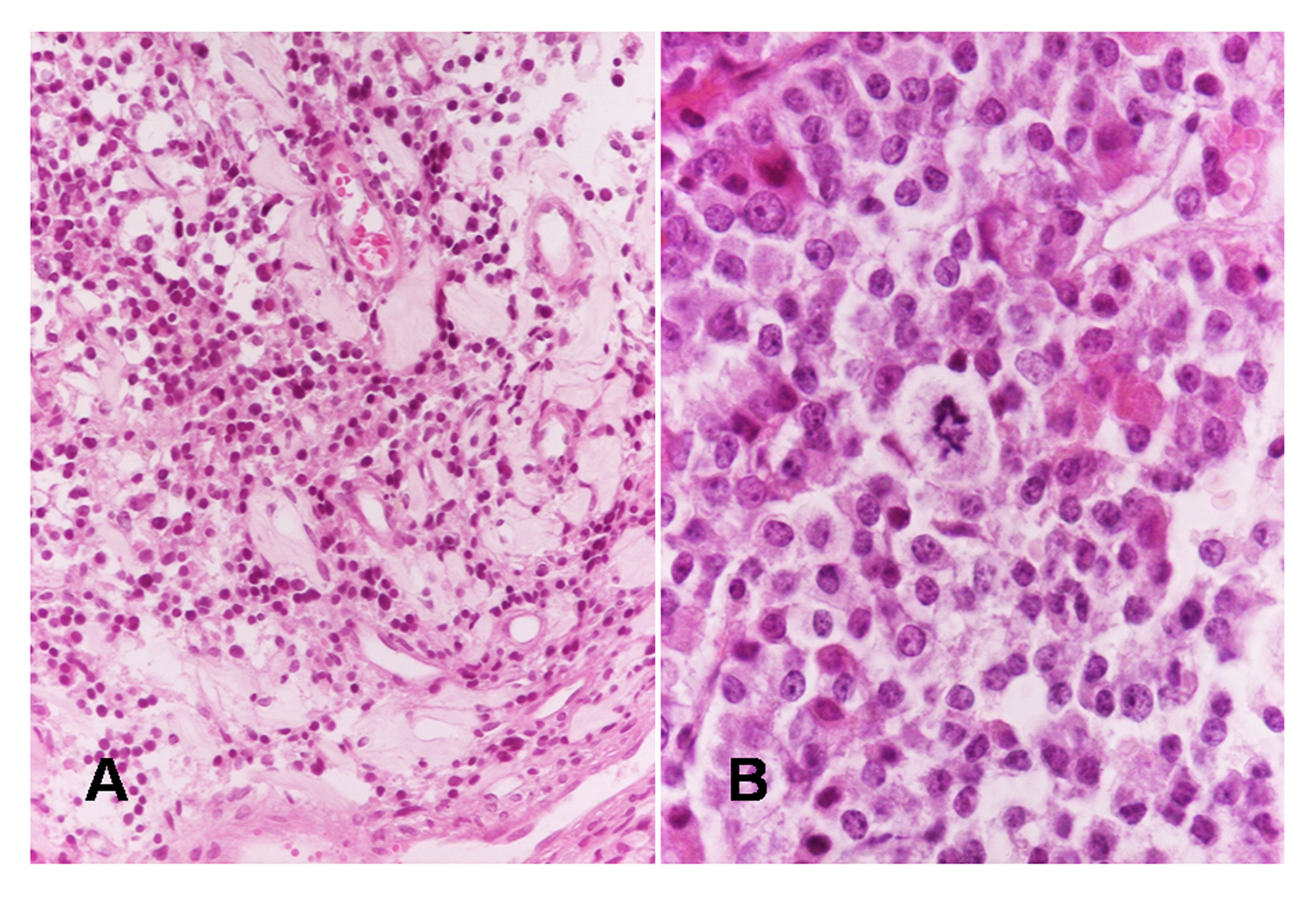


**Figure S3** Histopathological features of the pituitary adenoma from patient VIII. The specimen, obtained following radiotherapy, shows hyperchromatic nuclei and perivascular fibrosis representing post-treatment changes (A, HE – x10). An atypical mitosis is also present in this case (B, HE – x40)

**Figure S4** Ultrastructural examination of XLAG adenomas shows DG somatotroph cells admixed with SG cells (A – x3000); SG cells with peripheral secretory granules, some showing juxtaposition to the cytoplasmic membrane (arrows) and abundant endoplasmic reticulum, features consistent with lactotrophs (B – x10000); SG somatotroph cell containing a fibrous body (asterisk) (C – x10000)

**Supplementary tables**

**Table S1 Breakpoint junction features of the rearrangements identified in seven subjects with duplications encompassing *GPR101***

| **Case** | **Rearrangement** | **Genomic interval (ChrX)** | **Breakpoint signature** |
| --- | --- | --- | --- |
| **I** | DUP-NML-INV/DUP | 135557805- 135640218, 136027160- 136224960 | 5 bp microhomology at Jct1, 8 bp microhomology at Jct2 |
| **II** | Tandem DUP | 135631918- 136138344 | 6 bp microhomology |
| **III** | Tandem DUP | 135605028-136221008 | *Alu-Alu* mediated rearrangement with 33 bp microhomology (*Alu*Y->*Alu*Y) |
| **IV** | Tandem DUP | 135601432- 136176374 | 2 bp microhomology |
| **V** | Tandem DUP | 135599808- 136291833 | 1 bp microhomology |
| **VI** | Tandem DUP | 135587056-136302873 | 4 bp microhomology |
| **VII** | Tandem DUP | 135605143- 136187364 | 21 bp insertion (CTTGGGCAAGGCCTTTAATGA) |
| **VIII** | Tandem DUP (mosaic) | 135596446-136254970 | 1 bp microhomology |

**Table S2 Clinical features of XLAG patients**. OGTT: oral glucose tolerance test, ULN: upper limit of normal, TSS: transsphenoidal surgery, TC: transcranial surgery, RT: radiotherapy (conventional), GK: gamma-knife, PEG-V: pegvisomant, DI: diabetes insipidus, PA: pituitary adenoma, NA: not available

| **Case** | **Sex** | **Country** | **Germline/**  **somatic** | **Age at onset (months)** | **Age at diagnosis (years)** | **GH** | **OGTT – GH levels** | **IGF-1 (xULN)** | **PRL (xULN)** | **Tumour/**  **hyperplasia size** | **Treatment** | **Histology** | **Disease controlled** | **Hypopituitarism (axis)** | **Current Age** | **Previously published** |
| --- | --- | --- | --- | --- | --- | --- | --- | --- | --- | --- | --- | --- | --- | --- | --- | --- |
| I | F | UK | Germline | 18 | 4.1 | 1150mU/l | Mean 1150mU/l | NA | 2 | Macroadenoma | Intrasellar yttrium implants x3, SSA | Mixed cell adenoma (acidophilic/ chromophobic) | Yes | Yes (ACTH, TSH, LH) | 50 | Yes [[8](#_ENREF_8),[10](#_ENREF_10)] |
| II | F | UK | Germline | 12 | 3.8 | 114mU/l | Nadir 47.8mU/l | 3.9 | <1 | Diffuse enlargement | SSA, SSA+DA, SSA, PEG-V | No surgery | Yes | No | 12 | No |
| III | F | Mexico | Germline | 24 | 12 | 103ng/ml | Paradoxical rise | 1.2 | NA | 13x12mm | TSS, GK, SSA+DA, DA | NA | Yes | Yes (TSH, LH) | 25 | No |
| IV | F | USA | Germline | 9 | 1.5 | 42ng/ml | Unchanged | 2.9 | 3.4 | Diffuse enlargement | DA, SSA, SSA+DA, hemihypophysectomy, SSA+DA, completion of hypophysectomy | Somatotroph, lactotroph and mammosomatotroph hyperplasia | Yes | Yes (ACTH, TSH, LH, GH, DI) | 30 | Yes [[9](#_ENREF_9)] |
| V | F | USA | Germline | 30 | 5.7 | 85.4ng/ml | NA | 3 | 3.7 | 12x13x15mm | TSS | PA, GH+ PRL+ | Yes | Yes (DI) | 8 | No |
| VI | F | India | Germline | 24 | 7 | 88.6ng/ml | Nadir 62.9ng/ml | 1.9 | 6.9 | 16x19x12mm | TSS, GK, SSA+DA | PA, GH+ PRL+ | Yes | No | 10 | No |
| VII | F | Canada | Germline | 21 | 2.8 | 38ng/ml | NA | 5 | 11.3 | 32x13x8mm | TSS, GK, SSA+DA | PA, GH+, PRL+ | Yes | No | 12 | No |
| VIII | M | Turkey | Somatic | 48 | 7 | NA | NA | NA | <1 | Macroadenoma | TSSx3, SSA+DA, TSS, RT, GK, PEG-V | PA, GH+ PRL+ | No | Yes (ACTH, TSH, LH) | 33 | No |
| IX | M | Canada | Somatic | 24 | 4.7 | >35ng/ml | Nadir 29ng/ml | 2 | 10.3 | 15x18x13mm | SSA+DA, SSA+DA+PEG-V | Somatotroph, lactotroph and mammosomatotroph hyperplasia | Yes | No | 11 | Yes [[11](#_ENREF_11)] |
| X | F | Germany | Germline | 15 | 2.7 | 60.3ng/ml | Paradoxical rise | 2.9 | 3.3 | 18x15x9mm | SSA+DA, TSS | PA, GH+ PRL+ | Yes | Yes (ACTH, TSH, GH, DI) | 6 | No |
| XI | F | UK | Germline | 36 | 3.5 | 1620mU/l | Nadir 1166mU/l | 2.2 | 65.7 | 24mm | SSA+DA, TC, RT, DA, DA+PEG-V | PA, GH+, PRL+ | Yes | Yes (ACTH, TSH, LH) | 12 | Yes [[12](#_ENREF_12),[13](#_ENREF_13)] |
| XII | F | Australia | Germline | 7 | 1.6 | 3000mU/l | NA | 3.9 | 11.3 | Macroadenoma | DA, TC | PA, GH+, PRL+ | Yes | Yes (ACTH, TSH, LH, GH, DI) | 15 | Yes [[1](#_ENREF_1),[13](#_ENREF_13)] |

**Table S3 Comparison of clinical features of XLAG patients with *AIP*pos and *GPR101*&*AIP*neg patients**. Ns: not significant

|  | **XLAG (n=12)** | ***AIP*pos (n=63)** | ***GPR101&AIP*neg (n=78)** | ***P* value**  **XLAG  vs  *AIP*pos** | ***P* value**  **XLAG**  **vs**  ***GPR101*&*AIP*neg** | ***P* value**  ***AIP*pos**  **vs**  ***GPR101*&*AIP*neg** |
| --- | --- | --- | --- | --- | --- | --- |
| **Females/total (%)** | 10/12 (83.3%) | 20/63 (31.7%) | 28/78 (35.9%) | **<0.001** | **<0.001** | Ns |
| **Age at onset, median [IQR]** | 1.9 years [1.1-2.4] | 15 years [12.5-15] | 15 years [11.2-15.7] | **<0.001** | **<0.001** | Ns |
| **Age at diagnosis, median [IQR]** | 4.4 years [2.7-6.7] | 16 years [13-20] | 18 years [14-23] | **<0.001** | **<0.001** | Ns |
| **Height standard deviation score (SDS), median [IQR]** | +5.4 [4-6.3] | +3.8 [2.4-4.9] | +3.4 [2.7-4.3] | **<0.05** | **<0.01** | Ns |
| **IGF-1 index, median [IQR]** | 2.9xULN [2-3.9] | 2xULN [1.2-2.7] | 2.3xULN [1.5-3.4] | Ns | Ns | Ns |
| **Hyperprolactinemia (%)** | 10/12 (83.3%) | 8/34 (23.5%) | 10/31 (32.3%) | **<0.001** | **<0.01** | Ns |
| **Maximum tumour diameter, median [IQR]** | 18 mm [14-25.5] | 22.5 mm [15.2-38.7] | 24.5 [16.5-41.5] | Ns | Ns | Ns |
| **Giant adenomas (>40 mm) (%)** | 0/12 | 6/24 (25%) | 10/34 (29.4%) | Ns | **<0.05** | Ns |
| **Pituitary hyperplasia (%)** | 3/12 (25%) | 1/38 (2.6%) | 0/34 | **<0.05** | **<0.01** | Ns |
| **Suprasellar extension (%)** | 11/12 (91.7%) | 20/28 (71.4%) | 17/26 (65.4%) | Ns | Ns | Ns |
| **Cavernous sinus invasion (%)** | 3/12 (25%) | 10/28 (35.7%) | 11/28 (39.3%) | Ns | Ns | Ns |
| **Pituitary apoplexy (%)** | 0/12 | 8/55 (14.5%) | 2/70 (2.9%) | Ns | Ns | **<0.05** |
| **Number of treatments, median [IQR]** | 3.5 [2-4.7] | 2 [1-4] | 3 [1-4] | Ns | Ns | Ns |
| **Hypopituitarism (%)** | 8/12 (66.7%) | 13/28 (46.4%) | 11/19 (57.9%) | Ns | Ns | Ns |

**Supplementary references**

1. Trivellin G, Daly AF, Faucz FR, Yuan B, Rostomyan L, Larco DO, Schernthaner-Reiter MH, Szarek E, Leal LF, Caberg JH, Castermans E, Villa C, Dimopoulos A, Chittiboina P, Xekouki P, Shah N, Metzger D, Lysy PA, Ferrante E, Strebkova N, Mazerkina N, Zatelli MC, Lodish M, Horvath A, de Alexandre RB, Manning AD, Levy I, Keil MF, Sierra Mde L, Palmeira L, Coppieters W, Georges M, Naves LA, Jamar M, Bours V, Wu TJ, Choong CS, Bertherat J, Chanson P, Kamenicky P, Farrell WE, Barlier A, Quezado M, Bjelobaba I, Stojilkovic SS, Wess J, Costanzi S, Liu P, Lupski JR, Beckers A, Stratakis CA. Gigantism and acromegaly due to Xq26 microduplications and GPR101 mutation. N Engl J Med. 2014. 371:2363-2374. 10.1056/NEJMoa1408028.

2. Yuan B, Harel T, Gu S, Liu P, Burglen L, Chantot-Bastaraud S, Gelowani V, Beck CR, Carvalho CM, Cheung SW, Coe A, Malan V, Munnich A, Magoulas PL, Potocki L, Lupski JR. Nonrecurrent 17p11.2p12 Rearrangement Events that Result in Two Concomitant Genomic Disorders: The PMP22-RAI1 Contiguous Gene Duplication Syndrome. Am J Hum Genet. 2015. 97:691-707. 10.1016/j.ajhg.2015.10.003.

3. Hastings PJ, Ira G, Lupski JR. A microhomology-mediated break-induced replication model for the origin of human copy number variation. PLoS Genet. 2009. 5:e1000327. 10.1371/journal.pgen.1000327.

4. Lee JA, Carvalho CM, Lupski JR. A DNA replication mechanism for generating nonrecurrent rearrangements associated with genomic disorders. Cell. 2007. 131:1235-1247. 10.1016/j.cell.2007.11.037.

5. Zhang F, Khajavi M, Connolly AM, Towne CF, Batish SD, Lupski JR. The DNA replication FoSTeS/MMBIR mechanism can generate genomic, genic and exonic complex rearrangements in humans. Nat Genet. 2009. 41:849-853. 10.1038/ng.399.

6. Gu S, Yuan B, Campbell IM, Beck CR, Carvalho CM, Nagamani SC, Erez A, Patel A, Bacino CA, Shaw CA, Stankiewicz P, Cheung SW, Bi W, Lupski JR. Alu-mediated diverse and complex pathogenic copy-number variants within human chromosome 17 at p13.3. Hum Mol Genet. 2015. 24:4061-4077. 10.1093/hmg/ddv146.

7. Mayle R, Campbell IM, Beck CR, Yu Y, Wilson M, Shaw CA, Bjergbaek L, Lupski JR, Ira G. DNA REPAIR. Mus81 and converging forks limit the mutagenicity of replication fork breakage. Science. 2015. 349:742-747. 10.1126/science.aaa8391.

8. Rosenstock J, Doyle FH, Hall R, Mashiter K, Joplin GF. Childhood acromegaly successfully treated with interstitial irradiation using yttrium-90. Acta Paediatr Scand. 1982. 71:851-855.

9. Moran A, Asa SL, Kovacs K, Horvath E, Singer W, Sagman U, Reubi JC, Wilson CB, Larson R, Pescovitz OH. Gigantism due to pituitary mammosomatotroph hyperplasia. N Engl J Med. 1990. 323:322-327. 10.1056/NEJM199008023230507.

10. Lewis PD, Van Noorden S. Pituitary abnormalities in acromegaly. Arch Pathol. 1972. 94:119-126.

11. Rodd C, Millette M, Iacovazzo D, Stiles C, Barry S, Evanson J, Albrecht S, Caswell R, Bunce B, Jose S, Trouillas J, Roncaroli F, Sampson J, Ellard S, Korbonits M. Somatic GPR101 duplication causing X-linked acrogigantism (X-LAG) - diagnosis and management. J Clin Endocrinol Metab. 2016. 10.1210/jc.2015-4366.

12. Davies JH, Cheetham T. Investigation and management of tall stature. Arch Dis Child. 2014. 99:772-777. 10.1136/archdischild-2013-304830.

13. Beckers A, Lodish MB, Trivellin G, Rostomyan L, Lee M, Faucz FR, Yuan B, Choong CS, Caberg JH, Verrua E, Naves LA, Cheetham TD, Young J, Lysy PA, Petrossians P, Cotterill A, Shah NS, Metzger D, Castermans E, Ambrosio MR, Villa C, Strebkova N, Mazerkina N, Gaillard S, Barra GB, Casulari LA, Neggers SJ, Salvatori R, Jaffrain-Rea ML, Zacharin M, Santamaria BL, Zacharieva S, Lim EM, Mantovani G, Zatelli MC, Collins MT, Bonneville JF, Quezado M, Chittiboina P, Oldfield EH, Bours V, Liu P, W WdH, Pellegata N, Lupski JR, Daly AF, Stratakis CA. X-linked acrogigantism syndrome: clinical profile and therapeutic responses. Endocr Relat Cancer. 2015. 22:353-367. 10.1530/ERC-15-0038.
